# The Bangladesh road traffic sign dataset in real-world images for traffic sign recognition

**DOI:** 10.1016/j.dib.2025.111523

**Published:** 2025-03-27

**Authors:** Md. Ariful Islam, Dewan Md. Farid

**Affiliations:** Department of Computer Science and Engineering, United International University United City, Madani Avenue, Badda, Dhaka 1212, Bangladesh

**Keywords:** Traffic sign dataset, Traffic sign detection, Traffic sign classification, Image processing, Vehicle safety

## Abstract

Traffic sign detection and classification have significant impacts in the field of automated driving system, traffic management, driver assistance system, to detect traffic rules violations etc. In this paper, we have presented the Bangladesh road traffic sign benchmark dataset, which consists of 10259 real-world traffic sign images captured from various locations in Bangladesh and 10259 annotated images. A Total of 31 distinct traffic sign images were collected including Crossroad, Emergency Stopping, Sharp left turn. For image annotation, a sophisticated tool, Roboflow, has been utilized and data augmentation techniques have been applied to enhance the diversity of the images. The dataset is useful for training and testing of any deep convolutional neural networks (CNNs) models for traffic sign recognition. The dataset is publicly accessible via the following link: https://zenodo.org/records/14969122.

Specifications Table

**Table:** 

Subject	Computer Vision and Pattern Recognition
Specific subject area	Traffic Sign Detection and Classification, Image Processing
Data format	Raw digital images (.jpg) Image annotation values (.txt)
Type of data	2D-RGB image (JPG); TXT file (Image annotation values)
Data collection	Real-world Traffic sign images are collected using Realme 8 (resolution: 2608 × 4624 pixels) smartphone of 64-megapixel camera with lens specification of f/1.8, 26mm (wide) from various locations of Bangladesh. After collecting all the images, labelling and resizing was performed. Images are resized to 640 × 640 pixels.
Data source location	United International University, Dhaka, Bangladesh.
Data accessibility	Repository name: The Bangladesh Road Traffic Sign Dataset in Real-World Images Data identification number: 10.5281/zenodo.14969122 Direct URL to data: https://zenodo.org/records/14969122

## 1 Value of the Data

• The Bangladesh Road Traffic Sign Datasets can be used for training and testing Convolutional Neural Networks (CNN) models with Transfer Learning for traffic sign detection and classification.
• Researchers and developers working in the fields of machine learning, computer vision, and autonomous driving can significantly benefit from this dataset [1]. Additionally, government agencies and transportation departments can use this dataset to improve traffic management systems. Companies developing navigation systems, self-driving cars, and traffic monitoring solutions can also leverage this dataset to enhance their technologies.
• There is a total of 10259 images in this dataset, representing 31 different traffic signs in Bangladesh. By gathering more images per class or collecting some more varieties of traffic signs from other locations, the dataset can be extended. This extension may help to increase the accuracy of any machine learning or deep learning models in detecting traffic signs more effectively [2].
• This dataset can also be used in cross-domain research to study the applicability and generalizability of models across different geographic regions. Additionally, leveraging this dataset might help to develop IoT infrastructure to track and identify areas with frequent traffic rule violations, as well as to build an improved smart city infrastructure.
• Future work could tackle the use of multimodal models that integrate both visual and text information to enhance the identification of Bengali text-based traffic signs with the promise of greater accuracy in real-world implementation.

## 2 Background

Traffic signs play a vital role for ensuring road safety, guiding drivers, and minimizing road accidents. With the rapid rise autonomous driving technology and intelligent transportation system, accurate traffic sign detection is one of the most salient research areas in computer vision and deep learning. There are several well-known traffic sign datasets have been introduced such as the German Traffic Sign Recognition Benchmark (GTSRB) and the European Traffic Sign Dataset, which are widely used for training machine learning models. But these data set primarily represent traffic signs from western countries, which is different in the context of design, language and regulatory standards compared to Bangladeshi road signs.

Traffic signs in Bangladesh consist of Bengali letters and special symbols, making existing datasets inadequate to train deep learning models to recognize these local signs. In addition, most traffic signs in Bangladesh are not well maintained, become faded or obscured, making it even harder for automated recognition and classification. Although there are a few Bangladeshi traffic sign datasets exist, they are either small in size or lack diversity to ensure robust machine learning applications. To address this gap, we introduce the Bangladesh Road Traffic Sign Dataset (BTSD), a large-scale real-world traffic sign image dataset captured in various environmental conditions. This dataset lays the foundation for developing robust traffic sign detection and recognition models tailored to Bangladesh and, eventually, improved road safety, intelligent transport systems, and autonomous vehicle deployments.

## 3 Data Description

**Fig. 1:**
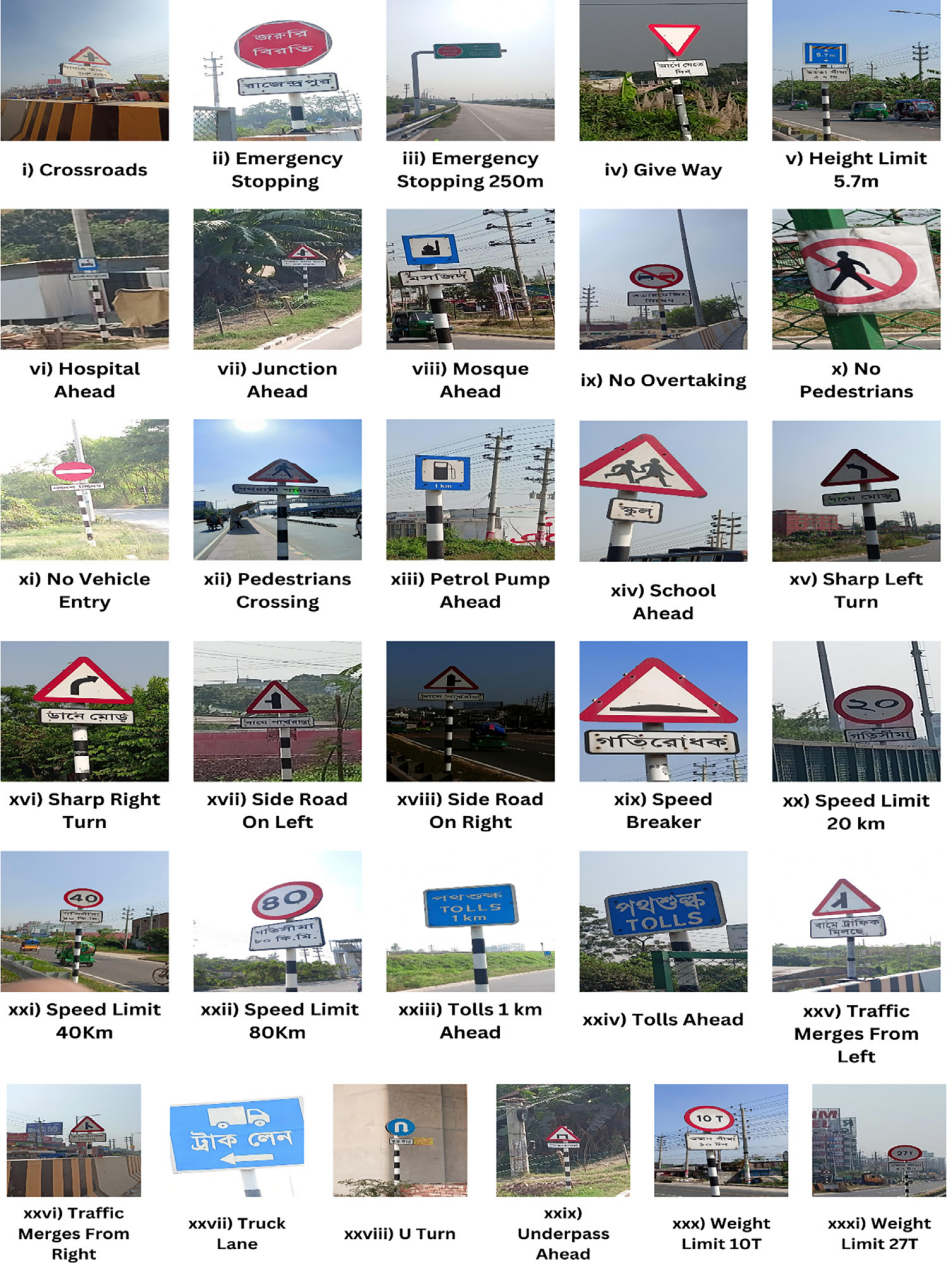
Sample images of the Bangladesh road traffic sign dataset.
ii. Annotated Images: The dataset also contains 10259 annotated image files, separated into test, train, and validation folders. These files list the precise locations of the objects, which are labelled in the corresponding images. The annotation has been performed manually using the Roboflow software, and the annotated values are stored in txt files. Fig. 2 provides two different annotated image files.

Traffic sign provides useful information, warning and regulations to road users for frequent and safe traffic system and reducing accidents. Systematic development and analysis of traffic safety sign may help to reduce traffic accidents [3]. This traffic sign dataset is an image dataset of Bangladeshi traffic signs where there are 31 different classes. The classes include: (i) Crossroads; (ii) Emergency Stopping; (iii) Emergency Stopping 250m; (iv) Give Way; (v) Height Limit 5.7m; (vi) Hospital Ahead; (vii) Junction Ahead; (viii) Mosque Ahead; (ix) No Overtaking; (x) No Pedestrians; (xi) No Vehicle Entry; (xii) Pedestrians Crossing; (xiii) Petrol Pump Ahead; (xiv) School Ahead; (xv) Sharp Left Turn; (xvi) Sharp Right Turn; (xvii) Side Road On Left; (xviii) Side Road On Right; (xix) Speed Breaker; (xx) Speed Limit 20 km; (xxi) Speed Limit 40Km; (xxii) Speed Limit 80Km; (xxiii) Tolls 1 km Ahead; (xxiv) Tolls Ahead; (xxv) Traffic Merges From Left; (xxvi) Traffic Merges From Right; (xxvii) Truck Lane; (xxviii) U Turn; (xxix) Underpass Ahead; (xxx) Weight Limit 10T; and (xxxi) Weight Limit 27T. Data has been stored in Zenodo. In the dataset, there are two types of data files, which are: (i) Raw Images; and (ii) Annotated Images.

i. Raw Images: The dataset consists of 10259 raw traffic sign images taken from various locations in Bangladesh, capturing a wide range of angles, visibility conditions, and atmospheric variables, using a smartphone camera. In the dataset, all images are in JPG format. A few representative images of the dataset have been depicted in Fig. 1 for all classes.

**Fig. 2:**
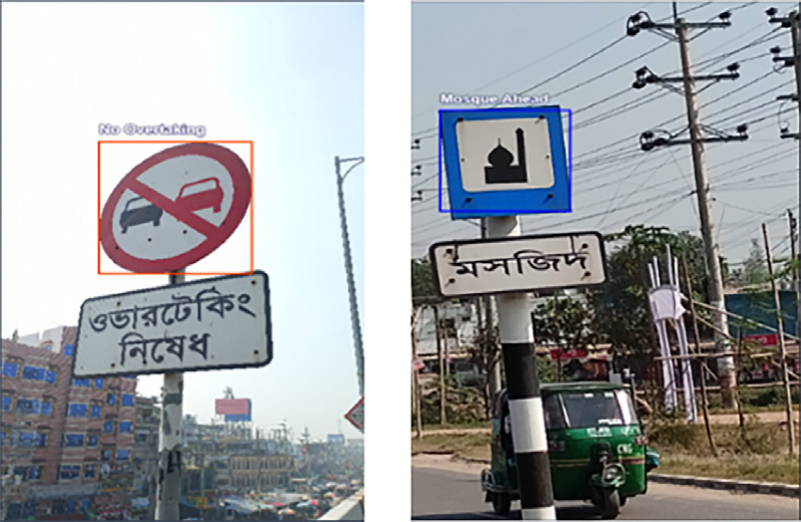
Sample image of manually annotated images. Left: No Overtaking; Right: Mosque Ahead.

The number of images per class with other details is given in Table 1. There are multitudes of available datasets to train deep learning models, such as the European Dataset [4], the German Traffic Sign Recognition Benchmark [5], the Bangladeshi Traffic Sign Dataset [6], the Indian Traffic Sign Detection Dataset [7], Traffic Sign in Bangladesh [8], etc. A simple comparison of these public vehicle datasets with the BTSD dataset is given in Table 2. Most of these datasets are from western countries, which differ in language, structure, and regulatory standards. These differences may make them inappropriate for use in Bangladesh. While there are some traffic sign datasets available from Bangladesh, these are small in size and contain fewer classes. BTSD, however, offers more diversified data with greater numbers of real-world traffic sign images captured from various locations around the country. In addition, the dataset includes images with obstacles, and varying environmental conditions to ensure that machine learning models can be trained for real-world scenarios, making it a valuable resource for robust traffic sign recognition in Bangladesh.

**Table 1:** Data description of the Bangladesh road traffic sign dataset.

Classes	No. of raw images	No. of annotated images	Total no. of images
Crossroads	94	94	188
Emergency Stopping	142	142	284
Emergency Stopping 250m	175	175	350
Give Way	155	155	310
Height Limit 5.7m	964	964	1928
Hospital Ahead	14	14	28
Junction Ahead	49	49	98
Mosque Ahead	587	587	1174
No Overtaking	526	526	1052
No Pedestrians	80	80	160
No Vehicle Entry	182	182	364
Pedestrians Crossing	606	606	1212
Petrol Pump Ahead	652	652	1304
School Ahead	277	277	554
Sharp Left Turn	323	323	646
Sharp Right Turn	360	360	720
Side Road On Left	853	853	1706
Side Road On Right	1069	1069	2138
Speed Breaker	40	410	450
Speed Limit 20 km	66	66	132
Speed Limit 40Km	598	598	1196
Speed Limit 80Km	428	428	856
Tolls 1 km Ahead	67	67	134
Tolls Ahead	58	58	116
Traffic Merges From Left	135	135	270
Traffic Merges From Right	22	22	44
Truck Lane	92	92	184
U Turn	75	75	150
Underpass Ahead	183	183	366
Weight Limit 10T	541	541	1082
Weight Limit 27T	476	476	952
TOTAL:	10259	10259	20518

**Table 2:** Comparison with others traffic sign datasets.

No.	Dataset	Number of Classes	Total Images
1	The European Dataset [4]	164	80000
2	Indian Traffic Sign Detection Dataset [7]	-	1264
3	Bangladeshi Traffic Sign Dataset [6]	15	2986
4	Traffic Sign in Bangladesh [8]	13	2000
5	German Traffic Sign Recognition Benchmark [5]	43	50000
6	The Bangladesh Road Traffic Sign Dataset in Real-World Images	31	10259

## 4 Experimental Design, Materials And Methods

The data collection, data pre-processing, and data annotation techniques to acquire the final datasets are discussed in this section.

### 4.1 Data collection

**Fig. 3:**
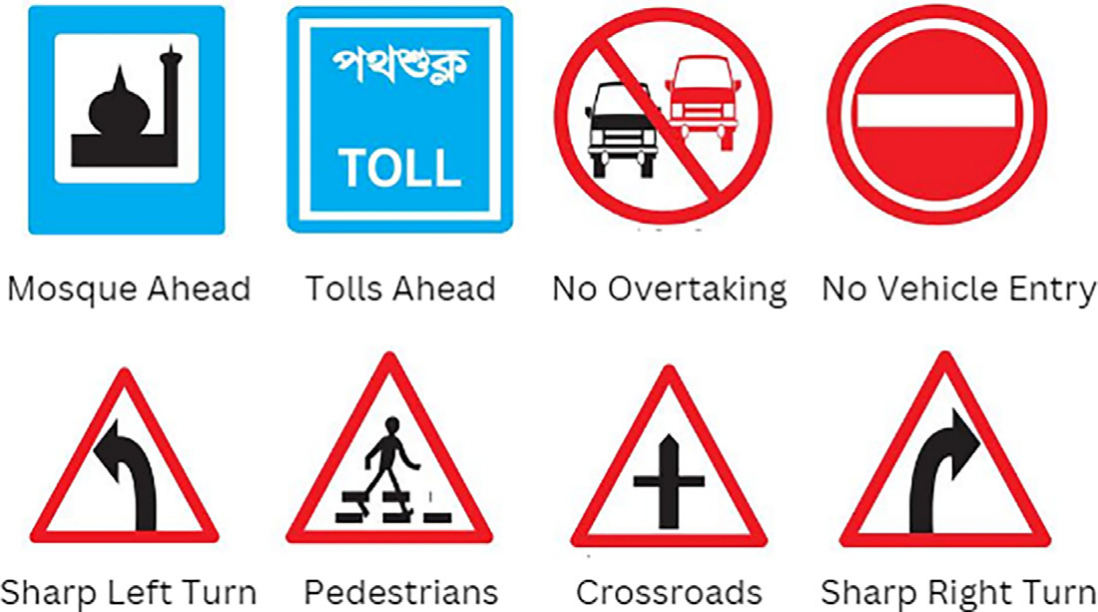
Sample image of BRTA traffic sign images.

Initially, the official dataset of Bangladesh traffic signs has been obtained from the Bangladesh Road Transport Authority (BRTA) website (https://bsp.brta.gov.bd/trafficDrivingTestGiudeline?lan=en). Fig. 3 illustrates a sample of this data. This official dataset has served as a reference for collecting traffic signs from various locations across Bangladesh. To create this dataset, we have utilized a REALME 8 smartphone's camera to capture images from different highways, expressways, and local roads. To enhance the variance of the data, diverse locations, viewing angles, weather conditions, scenarios, and distances have been considered.

### 4.2 Data annotation and processing

Following the data collection phase, we have ensured that all raw images have been converted to JPG format. Subsequently, we have copied all the raw images into a separate folder for annotation. An annotation file is a file that includes labels or annotations related to particular areas or objects in a picture [9]. These annotation data provide meaningful insights about the content of the image, such as location, size, etc. [10]. The images have been annotated using Roboflow (https://roboflow.com), a robust image annotation tool, in a format suitable for use with YOLOv9. During the annotation process, each image has been opened sequentially in the program. A rectangular shape has been manually created around the boundaries of each sign to accurately indicate its precise placement within the image using X-Y coordinates. Ultimately, a label has been assigned to every traffic sign. While performing the labelling process, Roboflow has stored the annotated values in TXT files. Subsequent to this, all image files have been resized to 640 × 640 pixels. The dataset has been divided into training, testing, and validation datasets in an 87:8:5 ratio. In the next step, data augmentation has been applied to the training dataset. Data augmentation is a common machine learning process used to increase the amount and diversity of data and mitigate overfitting in small datasets [11]. As part of the data augmentation process for the BTSD dataset, two new versions of each source image have been created by randomly varying the brightness from 0% to -25%. This process has yielded an additional 14033 images for the training data, but the augmented images have not been provided in the directory. The augmented images have also been saved in JPG format.

## Limitations

There are certain restrictions on the dataset. There are a total of 31 different classes, but the dataset does not include all the classes of traffic sign in Bangladesh. Besides these, there are some classes where the number of images is comparatively low. Collecting images in extreme weather conditions, such as foggy or rainy days, may enhance the dataset.

## Ethics Statement

The authors have follow the ethical requirements for publication in Data in Brief and confirming that the this work does not involve human subjects, animal experiments, or any data collected from social media platforms.

## CRediT Author Statement

**Md Ariful Islam:** Data collection, Data Annotation and Processing; Model validation, Writing – Original draft. **Dewan Md. Farid**: Conceptualization, Analytical reviewing, Supervision.

## Data Availability

zenodoThe Bangladesh Road Traffic Sign Dataset in Real-World Images (Original data)
